# The Membrane-Associated Form of α_s1_-Casein Interacts with Cholesterol-Rich Detergent-Resistant Microdomains

**DOI:** 10.1371/journal.pone.0115903

**Published:** 2014-12-30

**Authors:** Annabelle Le Parc, Edith Honvo Houéto, Natascha Pigat, Sophie Chat, Joëlle Leonil, Eric Chanat

**Affiliations:** 1 UR1196 Génomique et Physiologie de la Lactation, Institut National de la Recherche Agronomique, Jouy-en-Josas, France; 2 UMR1253 Science et Technologie du Lait et de l'Œuf, Institut National de la Recherche Agronomique, Rennes, France; University of British Columbia, Canada

## Abstract

Caseins, the main milk proteins, interact with colloidal calcium phosphate to form the casein micelle. The mesostructure of this supramolecular assembly markedly influences its nutritional and technological functionalities. However, its detailed molecular organization and the cellular mechanisms involved in its biogenesis have been only partially established. There is a growing body of evidence to support the concept that α_s1_-casein takes center stage in casein micelle building and transport in the secretory pathway of mammary epithelial cells. Here we have investigated the membrane-associated form of α_s1_-casein in rat mammary epithelial cells. Using metabolic labelling we show that α_s1_-casein becomes associated with membranes at the level of the endoplasmic reticulum, with no subsequent increase at the level of the Golgi apparatus. From morphological and biochemical data, it appears that caseins are in a tight relationship with membranes throughout the secretory pathway. On the other hand, we have observed that the membrane-associated form of α_s1_-casein co-purified with detergent-resistant membranes. It was poorly solubilised by Tween 20, partially insoluble in Lubrol WX, and substantially insoluble in Triton X-100. Finally, we found that cholesterol depletion results in the release of the membrane-associated form of α_s1_-casein. These experiments reveal that the insolubility of α_s1_-casein reflects its partial association with a cholesterol-rich detergent-resistant microdomain. We propose that the membrane-associated form of α_s1_-casein interacts with the lipid microdomain, or lipid raft, that forms within the membranes of the endoplasmic reticulum, for efficient forward transport and sorting in the secretory pathway of mammary epithelial cells.

## Introduction

During lactation, the mammary epithelial cells (MECs) synthesise and secrete substantial quantities of milk-specific proteins and other components such as lipids and lactose in a polarised fashion, from their apical surface into the alveolar lumen that they surround. Except in primates, the main milk proteins are the caseins, a family of acidic phosphoproteins (α_s1_-, α_s2_-, β- and κ-casein; for review see [1]). During their transport through the secretory pathway, caseins interact with calcium and calcium phosphate, and progressively self-aggregate to organize into a supramolecular structure, the casein micelle, which is released by exocytosis into the milk (see [2] and references therein). The chief physiological function of the casein micelle is supplying proteins, phosphate and calcium to neonates. In addition to its functional values, casein micelle production by the MEC is obviously of interest due to its economic importance for food industry.

Casein micelles have been the subject of research for decades, and disparate models of their internal structure have emerged, largely from morphological observations and biochemical and physical studies in vitro (for review see [3]). For many years, the hypothesis that caseins would be clustered into small spherical subunits that would be further linked together by calcium phosphate was widely accepted. This theory led to the submicelle model of the internal structure of the casein micelle. In recent years, models that refute the concept of discrete subunits within the casein micelle have emerged. One of these is the tangled web model, first proposed by Holt [4], and extended by Horne [5]. In the latter, caseins self-assemble primarily via electrostatic and hydrophobic forces to form a homogeneous network of casein polymers bound through interaction with calcium phosphate nanoclusters. Regardless of the model, k-casein which is highly glycosylated is believed to position preferentially near the micelle surface, forming the so-called outer hairy layer of k-casein at the protein-water interface, thereby stabilizing the structure and preventing it from aggregating. However, the detailed intrinsic organisation and the mechanisms involved in the formation of this structure have not been fully established. This is not trivial since it is well known that the mesostructure of the micelle determines the techno-functional characteristics of the milk protein fraction and impacts milk processing.

Casein micelles vary widely in size, compactness, and in protein and mineral composition across species, as well as occasionally among animals of the same species. The four major caseins are heterogeneous, their structural diversity being amplified in a given species due to genetic polymorphisms and variations in post-translational modifications. On the other hand, very little of the primary sequence of each of the caseins is fully conserved between species, making the caseins one of the most evolutionarily divergent families of mammalian proteins. Despite this high component heterogeneity, casein micelles are found in all mammalian milks as far as we know. Also, they seem quite similar at the ultra structural level [6]. Their structure as a whole is therefore believed to be analogous across species. Also, it has been reported that casein micelles form even in the absence of α_s1_- or ß-casein [7], [8]. Interactions between the various caseins and minerals during micelle biogenesis within the secretory pathway of the MEC might, therefore, involve rather the general physico-chemical and biochemical characteristics of these components. Of note, however, these characteristics are sufficiently specific to avoid direct incorporation of whey proteins into the native casein micelle.

Both biochemical [7], [9]–[12] and morphological [13], [14] information strongly suggests that aggregation of the caseins is initiated in the endoplasmic reticulum (ER) and gradually proceeds during their transport to the apical surface. We believe that we must exploit this spatio-temporal dimension of casein micelle biogenesis to obtain new insight about the intrinsic organization of the native casein micelle and the mechanisms implicated in their elaboration, and therefore study their construction within the secretory pathway of MECs. With this aim, we recently investigated the primary steps involved in casein interaction in the rough ER of both rat and goat MECs [15]. The highlights of this work are threefold. First, we have observed that the majority of both α_s1_- and ß-casein, which are cysteine-containing caseins in rat, was dimeric in the ER, as have suggested our previous study on milk caseins [10]. Second, non covalent interactions have also been observed in this compartment for the immature ER form of α_s1_-casein which is not phosphorylated (phosphorylation of the caseins occurs in the Golgi apparatus). In contrast, immature ß-casein is poorly aggregated in the ER. Finally, our experiments reveal the existence of a membrane-associated form of α_s1_-casein within the secretory pathway of MEC. We have found that protein dimerization via the disulphide bond greatly potentialize this interaction of α_s1_-casein with the membranes. Of note, bovine α_s1_-casein, which does not contain cysteine, is also known to dimerize at physiological ionic strength and pH [16]. Since α_s1_-casein is required for the efficient export of the other caseins from the ER [7], we believe that its membranous form may play a key role in the early steps of casein micelle biogenesis and/or casein transport in the secretory pathway. This conclusion is also supported by the observation that modification of the relative proportion of α_s1_-casein due to low or lack of expression invokes the accumulation of the other caseins in the ER coupled to an ER stress response, notably signalled by an enhanced expression of ER-resident proteins [7], [17].

In the present study, we further investigate the molecular basis of α_s1_-casein association with membranes of the secretory pathway, with emphasis on its potential incorporation into detergent-resistant membranes (DRMs) microdomains [18], an interaction which may be a prerequisite for the formation, transport and sorting of casein micelle to the apical plasma membrane domain of the MEC.

## Materials and Methods

### Animals and antibodies

Wistar rats raised in our institute (Nutrition et Régulation Lipidique des Fonctions Cérébrales, INRA, Jouy-en-Josas, France) were euthanatized by decapitation at mid-lactation. Animal welfare and conditions for animal handling were in accordance with the guideline of the European Community for experimental animal use (Directive n° 86/609/EEC) and experiments were approved by the French Ministry of Agriculture and Forestry and the Ministry of Research and Technology (agreement n° A78725). E. Chanat owns an accreditation for animal experimentation, level 1 (licence n° 78–62). Antibodies against mouse whole milk proteins (RAM/MSP) were obtained from Nordic Immunological laboratories (Tilburg, The Netherlands) and used at a dilution of 1∶2500 for immunoblotting. Rabbit polyclonal antibodies against calnexin (Cnx) were purchased from StressGen Biotechnologies Corp. (Victoria, BC, Canada) and used at a 1∶1000 dilution. Rabbit polyclonal antibodies against ERLIN2 (ER lipid raft associated 2) were from Sigma and used at 1∶2500 dilution. Mouse monoclonal antibody to protein disulfide isomerase (PDI) was from Enzo Life Sciences (clone 1D3) and used at 1∶5000 dilution. HRP-conjugated secondary antibodies were obtained from goat (anti-rabbit, Jackson Immunoresearch Lab., Inc., Avondale, PA, USA) or from sheep (anti-mouse, Sigma–Aldrich) and used at a dilution of 1∶5000 or 1∶2000, respectively. Unless otherwise indicated, chemicals were obtained from Sigma–Aldrich or Research Organics.

### Metabolic labelling

Metabolic labelling of mammary fragments was performed essentially as previously described [11], except that [^3^H]leucine was used. Briefly, dams were euthanatized and mammary glands were excised bilaterally and transferred to ice-cold 0.25 M sucrose. Samples were dissected from the connective tissue and muscles, finely chopped into ≈1–2 mm^3^ pieces using scissors on an ice-cold plastic pad, and further minced using a homemade multi-mounted razor blade device. Fragments were preincubated for 30 minutes in leucine-free DMEM under an atmosphere of 95% O_2_/5% CO_2_, and then pulse-labelled for 3 minutes in a small volume (5 ml/g of fragments) of leucine-free DMEM containing 1.85 MBq/ml (50 µCi/ml) L-[3,4,5-^3^H(N)]leucine (3,7 to 5,56 TBq/mmole, Perkin Elmer, Boston, MA, USA), all at 37°C. To end the labelling, fragments were quickly diluted in a 10-fold volume of pre-warmed complete DMEM and filtered on a Cell Strainer (100 µm, Becton Dickinson France, SA, Le Pont-de-Claix, France). To monitor the transport of labelled secretory proteins out of the ER, fragments were distributed into 20 ml Erlen (≈250 mg per experimental condition) containing 10 ml of complete DMEM and chased under gentle rotary shaking at 37°C, for the indicated times.

At the end of either the pulse or the chase, tissue fragments were poured into Cell Strainers which were transferred into 6-well cluster plates containing ice-cold Tris buffered saline (TBS, 25 mM Tris-HCl pH 7.4, 4.5 mM KCl, 137 mM NaCl, 0.7 mM Na_2_HPO_4_). Mammary fragments were extensively washed with ice-cold TBS and homogenised at 4°C in 1.5 ml 10 mM HEPES buffer pH 6.7 containing 250 mM sucrose, 1 mM EDTA, 1 mM magnesium acetate and an aliquot (10 µl/ml) of a protease inhibitor cocktail (Sigma-Aldrich), with three strokes of a tissue grinder (AA2 Teflon/glass, Thomas Scientific). The homogenate was centrifuged for 10 minutes at 1000 g, at 4°C, and the resulting supernatant, referred to as the post-nuclear supernatant (PNS), was collected. Protein concentrations in PNS were estimated. Aliquots of the PNSs (50 µg protein) were analysed by SDS-polyacrylamide gel electrophoresis (PAGE) followed by fluorography.

### Preparation of PNS and rough ER microsomal fraction

Mammary gland pieces were prepared with the use of scissors as above and washed 3 times for 10 minutes in 0.25 M sucrose at 4°C to remove milk constituents, and further minced using a homemade multi-mounted razor blade device. All subsequent steps were performed at 4°C. Tissue was homogenized in a 20 ml Teflon-glass homogenizer (BB, Thomas scientific) for 3 strokes. The homogenate was filtered through a piece of 150 µm polypropylene mesh (ZBF, Rüschlikon, Switzerland) and centrifuged at 8700 g in a Beckman JS 13.1 rotor for 13 minutes. The resulting supernatant is referred to as PNS. Membrane-bound organelles were sedimented from the PNS by centrifugation at 110,000 g for 1 hour. Total rough ER microsomes were prepared from the PNS by differential centrifugation followed by sucrose density gradient, as described by Paiement et al. for liver tissue [19], with the minor modifications reported in Le Parc et al. [15]. The final rough ER microsomal pellet was resuspended in 2 ml of 2 mM imidazole pH 7.4, 0.25 M sucrose, and aliquots were stored at −80°C. The rough ER microsomal fraction was previously characterized [15]. Protein concentration in the fractions was determined.

### Membrane and sucrose flotation gradient

All steps were performed at 4°C, and all buffers were supplemented with a protease inhibitor cocktail (Sigma-Aldrich). Aliquots of the microsomal fraction (100 µg protein) or of the membrane-bound organelles prepared by centrifugation (110,000 g for 1 hour) from the aliquots of PNS (100 µg protein) were diluted ≈5–10 fold (final volume: 250 µl) in either the absence of saponin in 10 mM Hepes pH 7.4 or in the presence of saponin (0.1%, w/v) in non-conservative conditions, i.e. a slightly basic pH (25 mM Hepes-KOH pH 7.4), the presence of a calcium chelator (20 mM EDTA) and salts (150 mM NaCl), plus a small quantity of mild detergent (0.3% v/v Tween 20) and of a reducing agent (5 mM DTT) or in carbonate buffer at pH 11.2 (100 mM Na_2_CO_3_ pH 11.2, 1 M KCl, 2 mM EDTA, 5 mM DTT). Samples were subjected to a 30 minutes incubation followed by centrifugation at 110,000 g for 1 hour. The resulting supernatants were collected and the membrane pellets were washed for 15 minutes in 10 mM Hepes pH 7.4 and pelleted as above. The carbonate supernatant was neutralised using 1 N HCl after the addition of a trace amount of phenol red. Proteins in supernatants were subjected to TCA precipitation (10% final concentration).

Membrane pellets were resuspended in 500 µl of HNE buffer (25 mM Hepes-KOH pH 7.4; 150 mM NaCl, 2 mM EDTA) and incubated for 30 minutes at 4°C. Samples were adjusted to 60% sucrose with 1.5 ml of 80% sucrose in HNE buffer and placed at the bottom of a Beckman Ultra-Clear SW55 TI tube and overlaid with 2 ml of 40% and 500 µl of 10% sucrose in HNE. Tubes were centrifuged for 15 hours at 35,000 g. Top to the bottom, fractions of 1 ml were collected; the volume of the last fraction was 500 µl. The pellet was solubilized in electrophoresis sample buffer, and the proteins in the fraction were subjected to TCA precipitation.

Proteins in the gradient fractions, in the gradient pellet, and in half of the above supernatant were analysed by SDS-PAGE followed by immunoblotting.

### Detergent lysis and differential centrifugation

All steps were performed at 4°C, and all buffers were supplemented with a protease inhibitor cocktail. Aliquots of the microsomal fraction (50 µg protein) or of the membrane-bound organelles prepared by centrifugation (110,000 g for 1 hour) from aliquots of PNS (50 µg protein) were diluted ≈5–10 fold (final volumes: 150 µl) in non-conservative buffer containing 0.1% (w/v) saponin. Samples were subjected to a 30 minutes incubation followed by centrifugation at 110,000 g for 1 hour. Membrane pellets were resuspended in HNE buffer, in the absence or in the presence of one of the following detergents: 1% Tween 20, 0.5% Lubrol WX (Lubrol 17A17, Serva) or 1% Triton X-100 (TX-100). Detergent concentrations are in weight-volume percentage. Samples were incubated for 30 minutes at 4°C and centrifuged as above. Proteins in the supernatant were subjected to TCA precipitation. Thirty percent of the pellet and supernatant were analysed by SDS-PAGE followed by either immunoblotting or Coomassie blue staining.

In some experiments, detergent resistant membrane pellets were subjected to a flotation sucrose gradient as described above. In this case, 2 (microsomes) or 4 (membrane-bound organelles prepared from PNS) times higher amount of material were analysed.

### Methyl-ß-cyclodextrin treatment

Aliquot of PNS or of the microsomal fraction were diluted at least 10 fold in ice-cold non-conservative buffer without Tween 20, supplemented with 10 µl of a protease inhibitor cocktail (Sigma-Aldrich), in the presence of the indicated concentration (0–50 mM) of methyl-ß-cyclodextrin (mßCD, Sigma-Aldrich). Samples were incubated for 30 minutes at 37°C and centrifuged at 110,000 g. Proteins in the resulting supernatant were subjected to TCA precipitation and the membrane pellet was directly solubilized in electrophoresis sample buffer before analysis by SDS-PAGE followed by immunoblotting.

### Electrophoresis, immunoblotting and quantification

Samples were analysed by SDS-PAGE using 12% gels according to Laemmli, except that the sample buffer contained 10 mM EDTA. Gels were stained, destained and processed for fluorography as previously described [20]. Immunoblotting was as described in Le Parc et al. [15]. The intensity of the ECL signal for relevant protein bands or the amounts of [^3^H]leucine-labelled caseins were quantified from X-ray film scans (300 dpi) using the ImageJ software (Wayne Rasband, NIH, USA, http://rsb.info.nih.gov/ij/). The background noise was estimated in the proximal area of the film and subtracted from the integrated density of the protein band. For PDI and ERLIN2, ECL signal was digitalised using ImageQuant LAS 4000 from GE Healthcare Life Sciences.

Protein concentrations were determined using the Peterson procedure [21] with bovine serum albumin as the standard.

For statistical analysis, we used the Friedman’s test.

### Electron microscopy

Tissues were fixed with 2% glutaraldehyde in 0.1 M Na cacodylate buffer pH 7.2, for 3 hours at room temperature. Samples were then postfixed with 1% osmium tetroxide containing 1.5% potassium cyanoferrate, gradually dehydrated in ethanol (30% to 100%) and embedded in Epon (Delta Microscopy, Labège, France).

Thin sections (70 nm) were collected onto 200 mesh cooper paladium grids, and counterstained with lead citrate before examination with a Zeiss EM 902 transmission electron microscope at 80 KV (MIMA2 - UR 1196 Génomique et Physiologie de la Lactation, INRA, plateau de Microscopie Electronique, 78352 Jouy-en-Josas, France). Microphotographies were acquired using MegaView III CCD camera and analysed with the ITEM software (Eloïse SARL, Roissy CDG, France).

## Results

### The membrane-associated form of α_s1_-casein is present in all compartments of the secretory pathway of MEC

**Figure 1:**
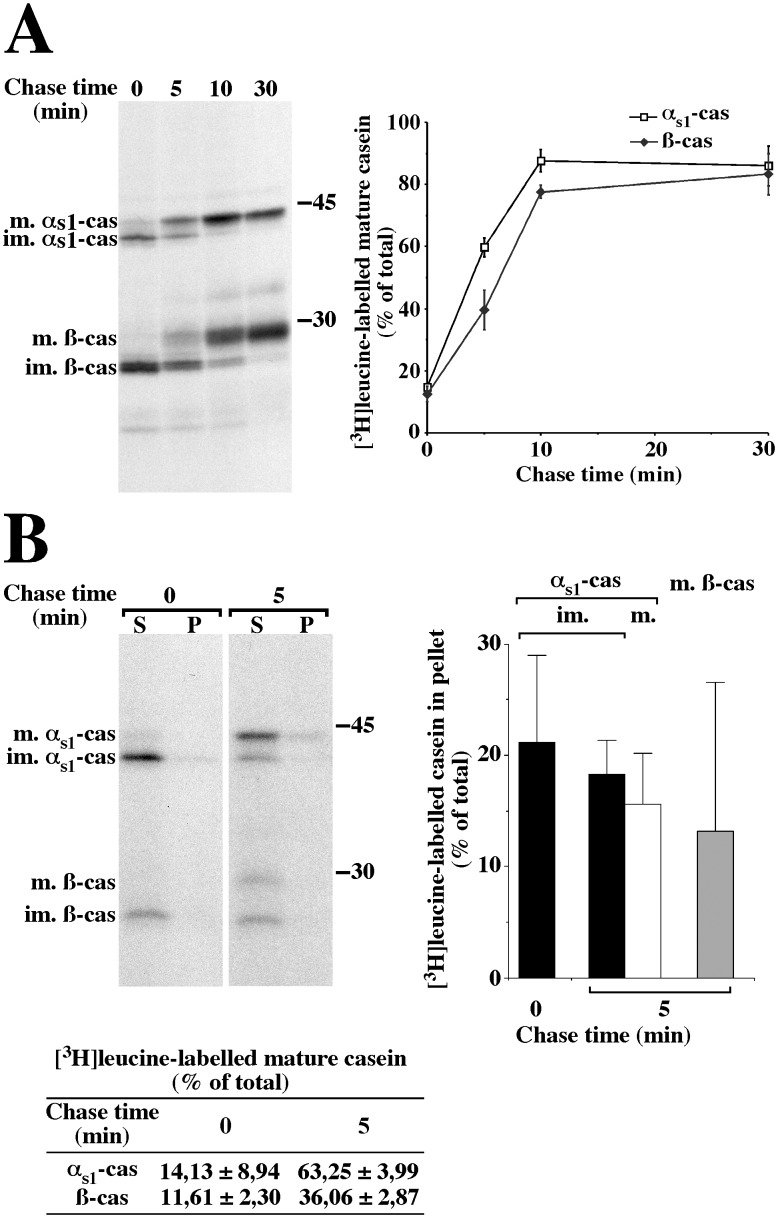
A membrane-associated form of α_s1_-casein is also present in the Golgi apparatus of rat MECs. (A) Time course for the arrival of newly synthesised caseins in the Golgi apparatus. Rat mammary gland fragments were pulse-labelled for 3 minutes with [^3^H]leucine and chased for the indicated times. At the end of the various chase periods, a PNS was prepared from the cells and analysed via SDS-PAGE and fluorography, followed by quantification of the immature (im.) and mature (m.) forms of both α_s1_- and ß-casein (cas). The amount of the mature form of the caseins was expressed as percent of total (sum of immature and mature forms). The mean ± s.d. from three independent experiments is shown. (B) Relative proportions of membrane-associated forms of the caseins in the ER and the Golgi apparatus. Rat mammary gland fragments were either pulse-labelled for 3 minutes with [^3^H]leucine or pulse-labelled and chased for 5 minutes. Aliquots of the PNS prepared from the cells were subjected to centrifugation and the resulting membrane pellet was resuspended and incubated for 30 minutes in non-conservative buffer in the presence of saponin. After centrifugation, supernatants (S) and pellets (P) were analysed via SDS-PAGE and fluorography, followed by quantification of the immature (im.) and mature (m.) forms of both α_s1_- and ß-casein. The amount of the mature form of the caseins (Table in panel B) was expressed as percent of total (sum of immature and mature forms). The amount of the various forms of the caseins in pellet (bar graph) is expressed as percent of total (sum of pellet and supernatant). The mean ± s.d. from three independent experiments is shown. Representative fluorograms are shown. Relative molecular masses (kDa) are indicated.

In our previous work, we showed the existence of a membrane-associated form of α_s1_-casein in the ER and in more distal compartments of the secretory pathway of MECs [15]. To better characterise this molecular form of α_s1_-casein and to obtain additional evidence for its existence in post-ER compartments, notably the Golgi apparatus, we used metabolic labelling coupled with SDS-PAGE analysis, on rat mammary tissue. The immature ER forms of the caseins, which are not yet phosphorylated, and the mature forms, which appear upon phosphorylation in the Golgi apparatus, can be easily resolved by SDS-PAGE [11], [15], [22]. Here, we chose [^3^H]leucine labelling to achieve direct quantitative comparison of α_s1_- and ß-casein (27 leucine residues in each). In order to investigate the membrane-associated form of α_s1_-casein in the Golgi apparatus, we first determined the kinetics of arrival of newly synthesised caseins in this compartment by monitoring their kinetics of maturation (Fig. 1A). Indeed, after a 3 minute pulse, newly synthesised caseins which were still in the ER were under their fast migrating immature forms. Conversion to their more slowly migrating forms occurred with a t½ of ≈4.0 minutes for α_s1_-casein and ≈6.5 minutes for ß-casein and maturation was virtually complete by 10 minutes of chase for the two proteins. These kinetics of maturation were slightly faster than that previously observed for rat casein labelled with [^35^S]methionine/cysteine mix [11]. On the other hand, the delay in the timing of the half-maturation of ß-casein, as compared to that of α_s1_-casein, is in agreement with previous data [11], [12], [22] and with our report showing that the phosphorylation of α_s1_-casein and ß-casein occurs in the Golgi apparatus and the trans Golgi network, respectively [9].

We then used this information to study the membrane-associated form of α_s1_-casein specifically that found in the ER and in the Golgi apparatus. With this aim, mammary gland fragments were either pulse-labelled for 3 minutes or pulse-labelled and chased for 5 minutes. Membrane-bound compartments prepared from the corresponding post-nuclear supernatant (PNS) were then permeabilised with saponin in non-conservative conditions, i.e. a slightly basic pH (pH 7.4), the presence of a calcium chelator and salts, plus a small quantity of mild detergent and of a reducing agent. We previously demonstrated that casein micelles are destroyed in these conditions and that only membrane-associated proteins, including the membrane-associated form of α_s1_-casein, are recovered in the membrane pellet after centrifugation [15]. As shown in Fig. 1B (Fig. 1B, table left panel), the proportion of total [^3^H]leucine-labelled mature caseins (supernatant plus pellet) measured for the two chase times after incubation with saponin in non-conservative conditions is very similar to that calculated directly from the PNS samples (Fig. 1A, graph). This result indicates that exposure to non-conservative conditions is not deleterious for one of the molecular forms of the two caseins. On the other hand, the relative amount of both immature and mature ß-casein is obviously much lower than that observed in the PNS (Fig. 1, compare autoradiograms). This was due to the fact that a relative high amount of ß-casein was released from membrane-bound organelle upon freeze/thawing of the PNS (data not shown). These results agree with our previous observation that ß-casein is mostly under soluble form in the early secretory pathway [15]. As expected, a non-negligible proportion of [^3^H]leucine-labelled immature α_s1_-casein (≈18–21% of total) remained associated with the membranous fractions after pulse or pulse followed by chase (Fig. 1B, right panel). These data are in agreement with our previous immunoblotting data (see Figure five A in [15]). After 5 minutes of chase, the proportion of [^3^H]leucine-labelled mature α_s1_-casein recovered with the membranous fraction (≈16% of total) was not significantly different to that of the immature form measured after pulse (Friedman’s statistical test). As for ß-casein, a single band at the level of the mature form was hardly discernible in the membrane pellet. These data confirmed that the association with membranes mainly concerns α_s1_-casein.

**Figure 2:**
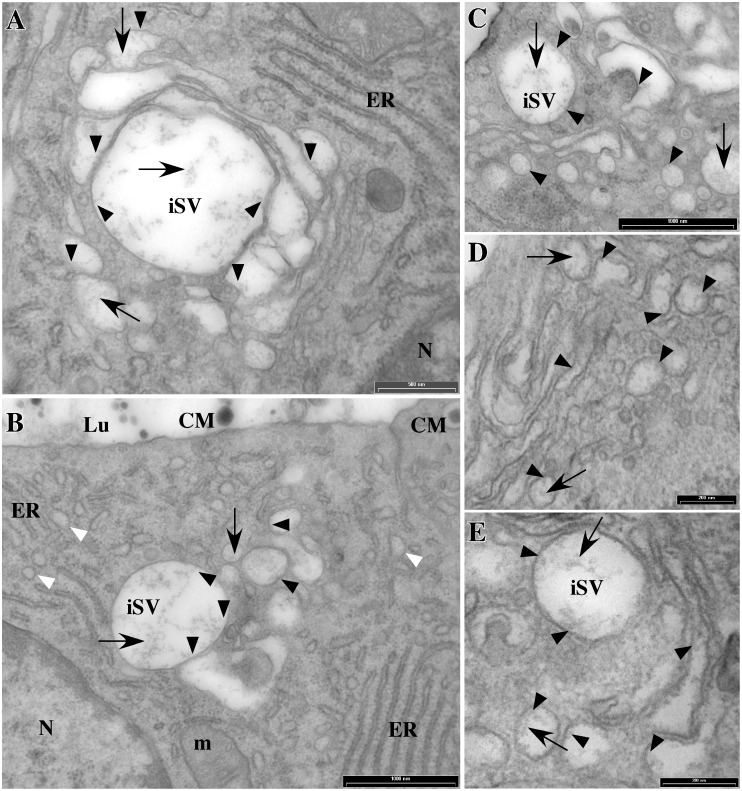
Appearance of the caseins in the Golgi region of lactating rat MECs. Mammary gland fragments from rat at mid-lactation were fixed and processed for electron microscopy. Golgi stacks, immature secretory vesicles (iSV) and other various distended elements of the Golgi region contain electron-dense particles loosely aggregated into interlaced structures or irregular linear clusters (arrows). These particles are also observed in distended rough ER components (see panel B, white arrowheads). Black arrowheads point to examples of close contact between electron-dense material and membranes of the compartments of the secretory pathway. Spherical compact casein micelles (CM) are found in mature secretory vesicles and in the lumen (Lu) of the acini (see panel B). N: nucleus; m: mitochondrion. Size of the bars is indicated.

Morphological analysis of rat MECs revealed that the premicellar casein aggregates that start to form in the Golgi apparatus, following phosphorylation of the caseins in this compartment, were often found to interact with the Golgi membrane via fine filamentous extensions (Fig. 2). Such particulates were already present in the less distended cis cisternae of the Golgi; they were either free in the lumen or in close interaction with the saccular membrane (Fig. 2A, C and D). As to the rough ER, the narrowness of its lumen, the greater concentration of electron-dense material in this compartment, and the fact that it is obviously more difficult to establish a link between this particulate material and what could be the first aggregates of immature caseins, did not allow us to draw any clear conclusion from these observations on the interaction of these proteins with the ER membranes, even in favourable areas where the ER was slightly dilated (Fig. 2B, white arrowheads). Of note, however, particulates were found to interact with the luminal leaflet of the membranes of purified rough ER microsomes [15].

**Figure 3:**
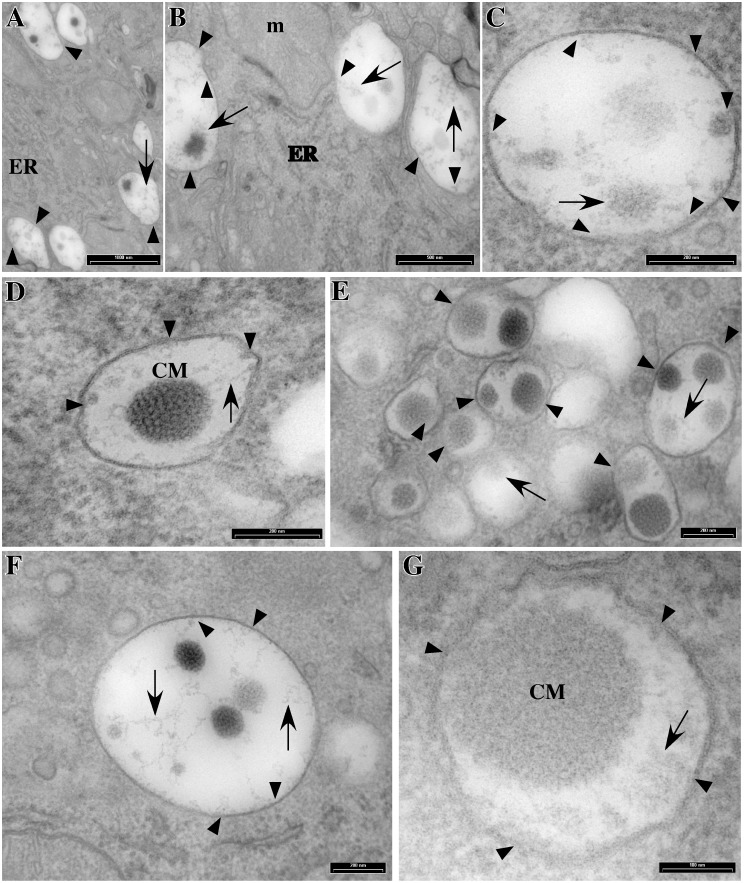
Appearance of the caseins in immature and mature secretory vesicles. Mammary gland fragments from rat at mid-lactation were fixed and processed for electron microscopy. Large aggregates of electron-dense particles are found in immature secretory vesicles (see A–C) together with interlaced structures and irregular linear clusters (arrows). Spherical compact aggregates presenting the typical honeycombed texture of casein micelles (CM) are observed in mature secretory vesicles (see D–G). Arrowheads point to examples of close contact between the electron-dense material of the interlaced structures or casein micelles and the membranes of the secretory vesicles. ER: endoplasmic reticulum; m: mitochondrion. Size of the bars is indicated.

Casein aggregates increase in size and become more compact in the trans Golgi cisternae or in newly-formed secretory vesicles (Fig. 2 and Fig. 3A–C), two compartments that are not easily distinguishable in the MECs. However, multiple examples of close contact between larger casein aggregates and the membranes of the immature vesicles were found (Fig. 3A–C). Casein aggregation further proceeds during vesicular transport to the apical cell surface, and casein micelles with their typical honeycomb appearance were present in mature secretory vesicles together with interlaced structures and irregular linear fine aggregates (Fig. 3D–G). Interestingly, the latter structures (Fig. 3D and G), as well as casein micelles, were also often seen in interaction with the vesicular membrane via root-like extensions of electron-dense material (Fig. 3 E–F). These observations, together with our biochemical data, suggest that caseins interact with the membranes of all compartments of the secretory pathway, possibly via the membrane-associated form of α_s1_-casein.

### α_s1_-Casein remains associated with a membrane fraction after extraction with non-ionic detergents

Having demonstrated the existence of a membrane-associated form of α_s1_-casein, a putative anchor for the association of casein aggregates with the membranes of the secretory pathway, we wished to determine the molecular basis of this interaction. With this aim, we investigated the possible resistance of the membrane-associated form of α_s1_-casein to membrane solubilisation with mild non-ionic detergents. Indeed, a correlation has been found between detergent-resistant membranes (DRMs) and membrane microdomains, or rafts, that are believed to play a key role in membrane traffic (for review see [23]). To investigate the possibility that α_s1_-casein interacts with DRMs, membrane-bound organelles were first subjected to permeabilisation by saponin in non-conservative conditions to remove soluble luminal proteins, and sedimented membranes were further extracted with detergents on ice. DRMs were prepared by centrifugation.

**Figure 4:**
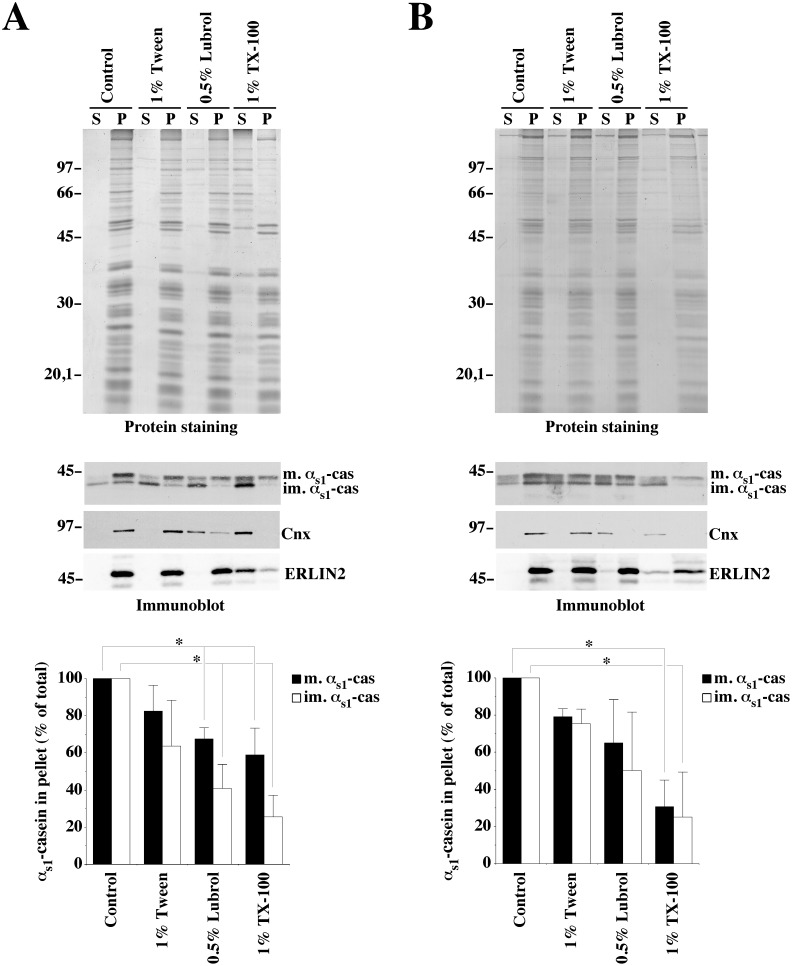
Comparison of membrane-associated- α_s1_-casein solubilities in various detergents. A purified rough microsome fraction (A) or membrane-bound organelles from a PNS (B) were incubated under non-conservative conditions in the presence of saponin and centrifuged. The resulting membrane pellets were resuspended in HNE buffer in the absence (Control) or in the presence of the indicated detergents, and incubated for 30 minutes at 4°C. After centrifugation, supernatant (S) and pellet (P) were analysed via SDS-PAGE followed by either Coomassie blue staining (Protein staining) or immunoblotting (Immunoblot) with antibodies against either mouse milk proteins, Cnx or ERLIN2. Immature and mature α_s1_-caseins were quantified by densitometry. For each condition, the amount of α_s1_-casein recovered in the supernatant under the control condition was subtracted from that measured under other conditions, and the proportion of the immature or mature form in the pellet was expressed as percent of the total (sum of pellet and supernatant). The mean ± s.d. from four independent experiments is shown. Detergent-treated samples were compared to control two-by-two for either immature or mature α_s1_-caseins using the Friedman’s test and statistical significance is indicated (*p<0.05). For Cnx and ERLIN2 representative immunoblots from two independent experiments are shown. Relative molecular masses (kDa) are indicated. im. α_s1_-cas: immature α_s1_-casein; m. α_s1_-cas: mature α_s1_-casein; TX-100: Triton X-100.

As shown in Fig. 4, some proteins were recovered in the supernatants with all detergents (Fig. 4, Protein staining), for both purified rough microsomes (Fig. 4A) and membrane-bound organelles prepared from PNS (Fig. 4B), but TX-100 was much more effective in disrupting lipid-protein interactions. In fact, with ER membranes, the proteins with a relative molecular mass greater than 50 kDa were quantitatively extracted by 1% TX-100. In most other cases, however, the vast majority of proteins was recovered in pellet, the pellets having very similar total protein patterns. The distribution of mature and immature α_s1_-casein in the detergent insoluble membrane pellet and supernatant was analysed and compared to the detergent resistance of a true transmembrane ER protein, namely calnexin (Cnx). The immunoblots show that, Cnx was not extracted by Tween 20 (Fig. 4, Immunoblot, Cnx) while a substantial proportion of α_s1_-casein, notably of the immature form, was recovered in the supernatant under these conditions. In contrast, Lubrol largely solubilized Cnx, whereas α_s1_-casein was still partly recovered in the membrane pellet. Finally, TX-100 further solubilised α_s1_-casein and entirely Cnx. These results with Cnx agreed with earlier observation [24]. As to ERLIN2 (ER lipid raft associated 2) which has been described as an ER lipid raft protein [25], it was recovered in pellet except with TX-100 treatment. Of note, ERLIN2 was better solubilised from purified microsomal membranes than when whole cell membranes were analysed. Concerning α_s1_-casein, we noticed a tendency to recover a smaller proportion of the immature form of the protein in the membrane fraction, as compared to the mature form. This differential recovery was more pronounced in the analysis of the rough microsomes where immature caseins predominate. One possible explanation for this finding is that the latter fraction contained a relative higher proportion of mature casein originating from contaminating casein micelles from milk than the purifying organelle fraction prepared from PNS, due to the procedure for the rough microsomes purification. However, as will be confirmed below, quantification clearly showed that, overall, the immature and mature forms of α_s1_-casein did not differ significantly (Friedman’s test) with respect to their resistance to detergent extraction (Fig. 4A and B, graph).

### The membrane-associated form of α_s1_-casein interacts with DRMs

**Figure 5:**
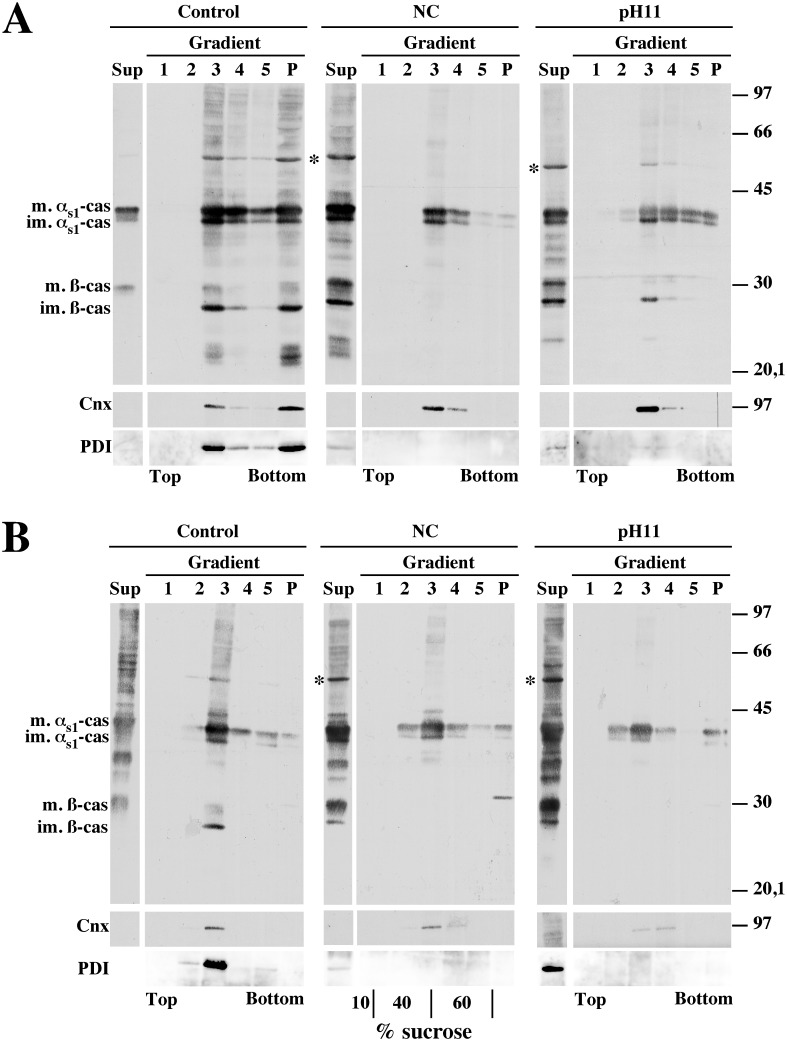
Purification of membrane-associated-α_s1_-casein fraction from rat mammary gland tissue on sucrose step gradients. A purified rough microsome fraction (A) or membrane-bound organelles from a PNS (B), both prepared from rat mammary gland tissue, were incubated in the absence (Control) or in the presence of saponin under non-conservative conditions (NC) or under carbonate buffer at pH 11.2 (pH 11). After centrifugation, supernatants were collected and membrane pellets were subjected to flotation on a sucrose step gradient (theoretical sucrose concentrations are indicated at the bottom of the central gel in panel B). Half of the supernatant (Sup), gradient fractions collected from the top (1 to 5) and gradient pellet (P) were analysed via SDS-PAGE followed by immunoblotting with polyclonal antibodies against either mouse milk proteins. Representative ECL signals from 5 (microsomes) or 3 (PNS) independent organelle preparations are shown. The distribution of Cnx and PDI was analysed within the above immunoblots. Relative molecular masses (kDa) are indicated. im. α_s1_-cas: immature α_s1_-casein; m. α_s1_-cas: mature α_s1_-casein; im. ß-cas: immature ß-casein; m. ß-cas: mature ß-casein; *: protein band with electrophoretic mobility identical to PDI.

To further investigate the possibility that the membrane-associated α_s1_-casein interacts with DRMs, we first developed an experimental procedure to analyse more specifically the content of subcellular membranes and of DRMs. We designed a sucrose density step gradient in which the membrane samples were adjusted to 60% sucrose and overlaid with 40 and 10% sucrose cushions (flotation in typical linear 40–5% sucrose gradient, e.g. [24], was not entirely satisfactory, see Fig. five in [15]). The top fractions 1–3 were the floating membrane fractions (see Fig. 5B, bottom of the central panel).

To validate this assay, we analysed the presence of the membrane-associated form of α_s1_-casein in membranes prepared from rough microsomes (Fig. 5A) or PNS-derived membrane-bound organelles (Fig. 5B) permeabilised under non-conservative conditions, or treated with carbonate at pH 11.2 to release the ribosomes and proteins which are not integral to the membranes [26], all in the presence of saponin and DTT. Without membrane permeabilisation, most of the milk specific proteins were recovered in the gradient fractions, notably with the membranes floating in fraction 3 and, for rough microsomes samples, also with those sedimenting in the gradient pellet (Fig. 5A, control). The relative distribution of membranes within the gradient was confirmed by the presence of Cnx in fraction 3, and in the gradient pellet with intact rough microsomes samples (Fig. 5, Cnx). In contrast, no Cnx was found in the gradient pellet after organelle permeabilisation and extraction (Fig. 5, NC and pH 11). The protein band putatively identified as protein disulphide isomerase (PDI, Fig. 5, asterisk; see the ECL signal that co-migrates with this band after immunoblotting with a monoclonal antibody against PDI) provided a convenient internal control for membrane permeabilisation. Indeed, this protein was totally recovered in the gradient under control conditions whereas most, if not all, was found in the supernatant after the membrane treatments. In line with this result, the large majority of immunoreactive proteins, including the caseins, was found in the supernatants (only half of the supernatants were analysed) after membrane permeabilisation or carbonate extraction at pH 11.2 (Fig. 5, NC and pH 11). As expected, however, a substantial proportion of α_s1_-casein was found with the floating membranes in fractions 2–3 of the gradients. In agreement with our previous report [15], ß-casein was not detected within the gradient fractions after saponin permeabilisation under non-conservative conditions. Note, however, that some was still present in fraction 3 or in the pellet after carbonate extraction at pH 11.2. Similar observation was made for PDI from microsomes, and higher amounts of α_s1_-casein were detected in the bottom fractions of the gradients. These results agreed with those in our previous report [15]. These experiments involving membrane flotation without detergent treatment definitively demonstrated the membrane association of α_s1_-casein.

**Figure 6:**
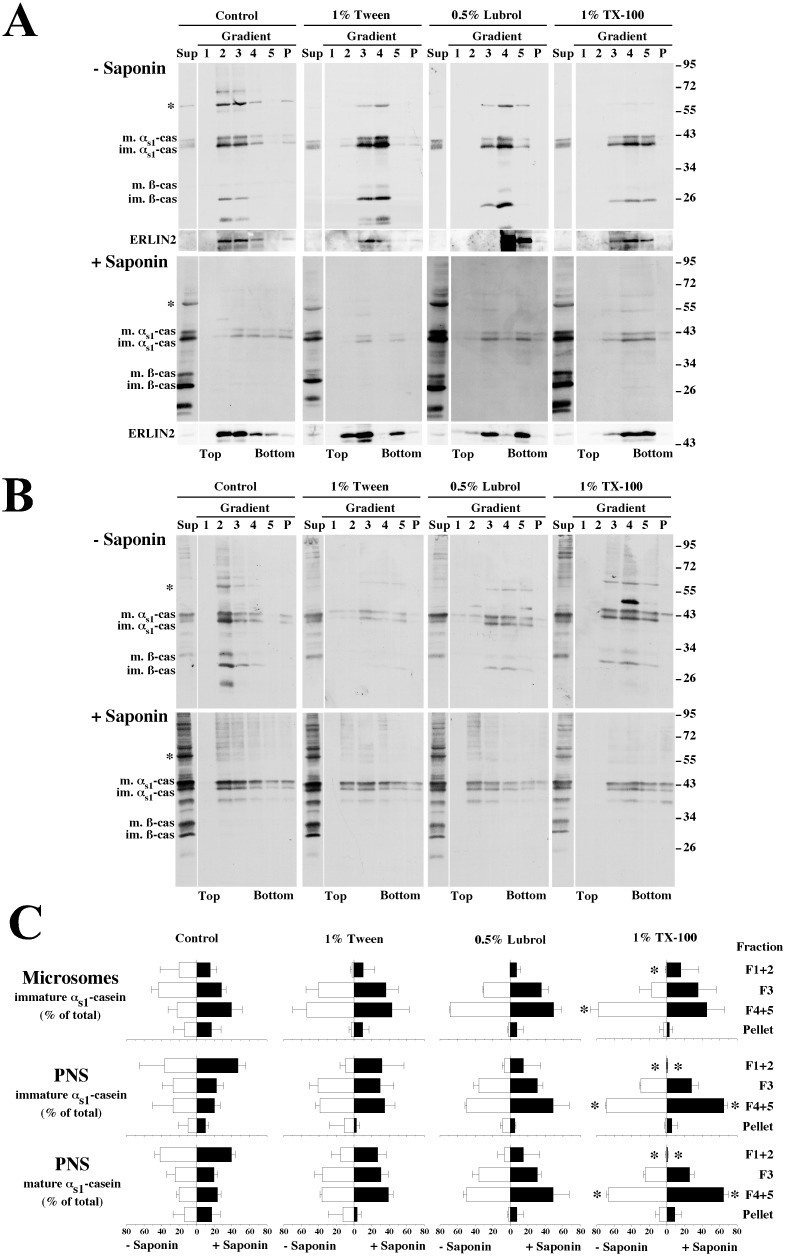
Membrane-associated-α_s1_-casein is associated with DRMs. A purified rough microsome fraction (A) or membrane-bound organelles from a PNS (B) were incubated in the absence of saponin (−Saponin) or under non-conservative conditions in the presence of saponin (+Saponin) and centrifuged. Supernatant was removed and membrane pellets were resuspended in HNE buffer, in the absence (Control) or the presence of the indicated detergents, and incubated for 30 minutes at 4°C. Detergent-treated membranes were subjected to flotation on a sucrose step gradient (sucrose concentrations as indicated in Fig. 5 panel B). Half of the supernatant (Sup), fractions collected from top to bottom (1–5) and gradient pellet (P) were analysed via SDS-PAGE followed by immunoblotting with an antibody against mouse milk proteins. Representative ECL signals from four experiments with three independent organelles preparations are shown. The distribution of ERLIN2 was analysed within the immunoblots shown in panel A. C. Quantification of membrane-associated-α_s1_-casein in DRMs. Immature (Microsomes), or immature and mature α_s1_-caseins (PNS) were quantified via densitometry. For each condition, the amounts of the indicated forms of α_s1_-casein recovered in the various fractions of the sucrose step gradient were measured and the proportion of the immature or mature forms of α_s1_-casein for each fraction was expressed as percent of the total (sum of gradient fractions and pellet). The means ± s.d. from four experiments with three independent organelles preparations are shown. The proportion of either immature or mature α_s1_-caseins in each fraction of the gradient from TX-100-treated samples was compared two-by-two to control data using the Friedman’s test and statistical significance is indicated (*p<0.05). Relative molecular masses (kDa) are indicated. im. α_s1_-cas: immature α_s1_-casein; m. α_s1_-cas: mature α_s1_-casein; im. ß-cas: immature ß-casein; m. ß-cas: mature ß-casein; TX-100: Triton X-100; *: protein band with electrophoretic mobility identical to PDI. F: fraction; TX-100: Triton X-100.

We then investigated whether the membrane-associated form of α_s1_-casein is associated with DRMs that are recovered as floating membranes in the sucrose step gradient (Fig. 6). As observed previously in Fig. 5 and confirmed here, milk proteins, including PDI, were recovered essentially in the light sucrose fractions (fractions 1–3), in the absence of saponin permeabilisation followed by incubation in control conditions (Fig. 6AB, Control, - Saponin). About 60–70% of immature or mature α_s1_-casein were found in these fractions (Fig. 6C). As expected, PDI as well as ß-casein were released from the lumen of the membrane-bound compartments when the membranes were first subjected to saponin permeabilisation (Fig. 6AB, Control, + Saponin). However, about two-thirds of immature or mature α_s1_-casein were again observed in the floating membrane fraction prepared from PNS (Fig. 6C). However, the proportion of immature α_s1_-casein in these fractions was reduced to ≈45% with microsomes, an increase of the proportion of the soluble form of this casein being clearly visible in fraction 4 plus 5. In these experiments, we also attempted to determine whether saponin treatment, which complexes membranous cholesterol, would influence membrane flotation and detergent extraction. Whereas its effect on membrane lipids was clearly highlighted by the release of soluble luminal proteins such as ß-casein and PDI (see above), it appeared not to significantly affect the relative distribution of membranes within the gradient fraction (Fig. 6A Control, ERLIN2). We interpreted the slight variations in the distribution as being largely a result of small deviations in the collection of fractions. For the same reasons, and because in numerous instances the distributions of α_s1_-casein appeared quite similar without or with saponin treatment, we have not yet determined the possible effect of saponin on the efficiency of extraction of membrane-associated α_s1_-casein by the different detergents (Fig. 6C, compare −Saponin and +Saponin).

In contrast, DRM preparation by flotation on sucrose step gradient lent support to the two effects of the detergent treatments. First, we confirmed the graded solubilisation of membrane-associated α_s1_-casein by Tween 20, Lubrol and TX-100. This finding is well documented in Fig. 6C, in which a gradual transition of membrane-associated α_s1_-casein from the light sucrose fractions containing DRMs under control conditions, namely fractions 1–3, toward the high-density fractions (4–5 and pellet) containing detergent solubilised α_s1_-casein clearly occurs. The differential distribution was statistically significant between control and TX-100 samples. Moreover, the relative efficiency of the extraction by these detergents appeared to be of the same order of magnitude as that observed by differential centrifugation in Fig. 4. The partial solubilisation of ERLIN2 by TX-100 was also confirmed. Second, our data show that the above detergents solubilised similar proportions of both the immature and mature forms of membrane-associated α_s1_-casein (Fig. 6C, compare immature and mature α_s1_-casein in PNS).

**Figure 7:**
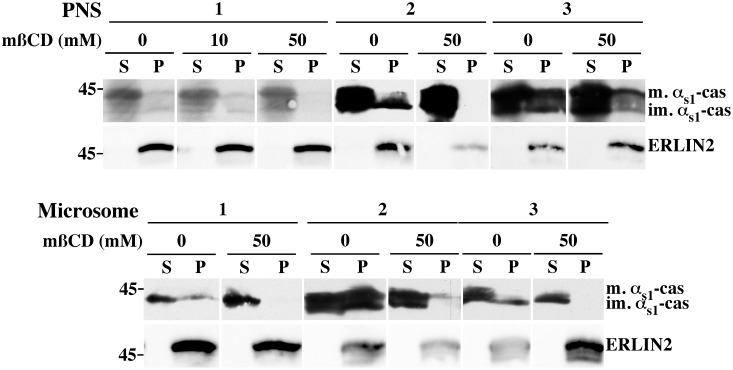
The DRMs containing α_s1_-casein are sensitive to cholesterol depletion. Membrane-bound organelles in PNS or purified rough microsomes fractions were incubated in non-conservative buffer without Tween 20 and saponin, in the absence or the presence of the indicated concentration of mßCD for 30 minutes at 37°C. After centrifugation, supernatant (S) and pellet (P) were analysed via SDS-PAGE followed by immunoblotting with antibodies against mouse milk proteins or ERLIN2. For each type of membranes, three independent experiments are shown. The protein concentration in the analysis of the PNS 1 was twice lower than for all other samples and most of the scans showing α_s1_-casein signal were taken from overexposed films for a better display of the large reduction of α_s1_-casein present in the membrane pellet after cholesterol extraction by mßCD. Relative molecular masses (kDa) are indicated. im. α_s1_-cas: immature α_s1_-casein; m. α_s1_-cas: mature α_s1_-casein.

If α_s1_-casein is associated with a DRM, the question arises whether cholesterol is needed to maintain its structure and/or DRM association of α_s1_-casein. To remove cholesterol from subcellular membranes, PNS or microsome samples were treated with methyl-ß-cyclodextrin (mßCD). When membranes were treated with 50 mM mßCD at 37°C, most, if not all α_s1_-casein was solubilized and recovered in the supernatant (Fig. 7). Consistent with the pioneer report of Browman et al. [25], ERLIN2 remained in the insoluble fraction in these conditions. We concluded from these results that both the immature and mature membrane associated forms of α_s1_-casein interact with DRMs.

## Discussion

Caseins are sorted to the apical domain of MEC for secretion. The current concept is that proteins destined for the apical or basolateral plasma membrane are sorted at the level of the trans-Golgi network on the basis of intrinsic sorting motifs (for review see [27]). We reasoned that, if the association of α_s1_-casein with membrane has anything to do with the sorting and/or the efficiency of casein transport in the secretory pathway, this interaction must be maintained, or even increased, in the Golgi apparatus. Our finding that the mature phosphorylated form of α_s1_-casein is also present in a membrane-associated form is consistent with this hypothesis [15]. To investigate this possibility further, we compared the behaviour of newly synthesised α_s1_- and ß-casein in the ER and in the Golgi apparatus, two steps of the secretory pathway that can be easily identified on the basis of casein phosphorylation/maturation. These experiments corroborated the differential behaviour of α_s1_- and ß-casein during the early steps of casein transport in the secretory pathway. First, we confirmed here that the phosphorylation of ß-casein is delayed as compared to that of α_s1_-casein as we, and others, have observed previously [9], [11], [12]. Secondly, and more importantly, we verified that ß-casein was highly soluble within both the ER and Golgi lumina, compared to α_s1_-casein. When whole PNS was analysed (Fig. 1A), the mean ratio of total α_s1_- to total ß-casein (immature plus mature forms) was 0.52±0.14. This is somewhat lower than the ratio that can be calculated from the casein content in the milk of mouse from published results [28]. However, the milk protein concentrations, as well as the relative proportions of the caseins, vary greatly not only among mouse species, but also among mouse strains [28], [29]. Moreover, reliable quantitative data on casein composition are absent for rat. After freeze/thawing of the PNS and centrifugation, we found a relative high amount of ß-casein in the resulting supernatant (data not shown), and the above mean ratio calculated for the caseins remaining in the membrane-bound organelle pellet was 2.07±0.60 (Fig. 1B), i.e. 75% of ß-casein is released from these compartments during sample processing because it is in a soluble form. Thirdly, we observed that the proportions of [^3^H]leucine-labelled immature (0 minute chase, ER form) and mature (5 minutes chase, Golgi form) α_s1_-casein recovered with the membranous fraction (≈16% of total, in agreement with our previous experiments using immunoblotting [15]) were not significantly different (Friedman’s statistical test). Altogether, these data indicate that the proportion of membrane-associated α_s1_-casein remains constant, at least between the ER and the Golgi apparatus. This consistency suggests the existence of an early sorting mechanism, prior to the maturation of caseins in the Golgi apparatus.

Clearly, α_s1_-casein is involved in the central stage of casein export from the ER. Possibly, its membrane-associated form plays a key role in casein transport and/or casein aggregation within the secretory pathway, where it might represent a nucleation anchor for casein micelle formation and/or a link molecule for the cytosolic secretion machinery. Pioneer studies concerning casein micelle formation involved transmission electron microscopy (for review see [14]), notably of rat mammary gland tissue (e.g. see [30]), and membrane connection of casein micelles was noticed early [31]. A more recent and thorough analysis of casein secretion in the mammary gland of rat also revealed the attachment of premicellar casein aggregates to membranes of the Golgi apparatus of rat MECs [13], but this observation has not yet been explained. At this stage, one cannot exclude the possibility that these short protein fibre strands are not native structures, but result from the processing of the samples for electron microscopy. Nevertheless, these images corroborate our biochemical analysis. In the present study, we clearly show that the connection of irregular linear clusters or of loose interlaced aggregates of caseins with the membranes of the Golgi apparatus, as well as of more mature casein micelle structures, with the membranes of the secretory pathway is not a rare event. We are confident that, although less obvious, such interactions also exist in the ER. Indeed, membrane-associated particulates were observed in the lumen of purified rough microsomes prepared from rat or goat MECs [15]. Others and we made similar observations in mice [32]–[34] and rabbit [35], [36]. Surprisingly, electron microscopy data on the formation of casein micelles in ruminants are scarce, both in cattle and goat. However, the association of casein aggregates with membranes was also observed in the latter species [7]. This result was consistent with our biochemical data, but we could not estimate whether the lower proportion of membrane-associated α_s1_-casein found in goat [15] correlated with fewer occurrences of casein-membrane interaction because the morphological approach do not allow for the reliable quantitation of them. Note, however, that such interactions were still observed in MECs that did not express α_s1_-casein [7], indicating that this casein is not exclusively responsible for the association of casein aggregates with membranes. In line with this, it should be noted that preliminary experiments with goat rough microsomes suggest that immature κ-casein behaves towards membranes much as immature α_s1_-casein does (unpublished data). Moreover, similar proportions of α_s1_- and κ-casein (≈5–7%) were found with the membrane pellet after rabbit MECs membrane extraction with carbonate at pH 11.2 (unpublished data). The latter finding, however, was not confirmed with the use of saponin permeabilisation in non-conservative conditions and, unfortunately, we do not yet have the immunological tools to analyse the behaviour of κ-casein in the rat experimental system. Moreover, κ-casein has three times less leucine, which made its quantification difficult in the present experiments using metabolic labelling. Given the foregoing and that κ-casein, in contrast to α_s1_-casein, is believed to position preferentially at the periphery of the micelle, we can not exclude that the association of α_s1_-casein with membrane is indirect and rather takes place through its interaction with a membranous form of κ-casein. However, investigation of the role of κ-casein in casein transport and casein micelle formation will be the subject of a separate study.

Cell membranes are partially resistant to solubilisation with mild non-ionic detergents in the cold. These DRMs are believed to be the biochemical remnants of the cellular lipid rafts [37]; they are enriched with cholesterol and sphingolipids [38]. Lipid rafts are thought to play a crucial role in the lipid-mediated sorting of cargo, notably at the trans-Golgi network, for their delivery to the cell surface (for review see [39]). Since the molecular interactions underlying the sorting of the caseins for exocytosis are unknown, including or not association of caseins with the membranes of the secretory compartments, it was important to determine whether they associate with lipid rafts on their way to the apical plasma membrane of MECs. We therefore ask whether they interact with DRMs. With the mild non-ionic detergents used in this study, we observed a gradation of α_s1_-casein solubilisation similar to that observed for other DRM marker proteins [24]. However, a substantial proportion of membrane-associated α_s1_-casein (25–30%) remained with DRMs prepared with TX-100. In striking contrast, we confirmed the solubilisation profile of Cnx, a transmembrane ER protein, being large with Lubrol and complete with TX-100. Since the mature casein present in the rough microsomes fraction appeared to be capable of better recovery in DRMs, compared to the immature form, we suspected that part of that signal might be a result of contaminating casein micelles. We therefore decided to prepare DRMs by flotation on sucrose gradients. The use of a linear sucrose gradient has proved unsatisfactory because MECs DRMs did not float as well as described by others using cell lines (e.g. see [24]), in particular when an analysis of the rough microsome samples was tried. This observation may have been largely due to the fact that MECs synthesize and secrete extremely large quantities of proteins during lactation. Thus, the membranes of the secretory pathway may be overloaded by proteins involved in protein synthesis and folding, ribosomes, and the secretory proteins themselves, preventing flotation using standard conditions. For MECs, cellular membranes or detergent extracts were therefore brought to 60% sucrose and were purified using flotation on a sucrose step gradient. Also noteworthy is the fact that the procedure involving saponin permeabilisation under non-conservative conditions was more effective to release proteins not integral to membranes than saponin in combination with carbonate treatment at pH 11.2 (Fig. 5). We also found that pretreatment of the membrane-bound compartments with saponin in non-conservative conditions was essential to avoid that a substantial part of the non-integral proteins remains trapped into the network of bilayered membranes and vesicular structures that results from detergents solubilisation [40]. The results obtained with this experimental system strongly suggested that the membrane-associated form of α_s1_-casein is associated to a DRM of MECs. Further evidence for the existence of a cholesterol-dependent DRM containing α_s1_-casein was obtained when membranes were treated with mßCD, which is known to selectively deplete biological membranes of cholesterol. Upon mßCD treatment at 37°C, sedimentation of α_s1_-casein with membranes was drastically decrease.

The sorting function for lipid microdomains in the cargo trafficking from the trans cisternae of the Golgi apparatus and the specific transport to the cell surface has been largely documented (for review see [39]). Our data are consistent with the occurrence of a membrane-associated form of α_s1_-casein interacting with the DRMs at an earlier stage of the secretory pathway, the cis Golgi or the ER, prior to casein maturation in the Golgi apparatus. The somewhat recent realisation that the sorting of, at least certain, secretory proteins occurs prior to exit from the ER is consistent with this hypothesis. Muniz et al., found that, in yeast, GPI-anchored protein markers are sorted from other secretory proteins in the ER, and packaged into distinct ER-derived vesicles for forward transport to the Golgi apparatus [41]. More recently, the characterisation of proteins enriched in lipid rafts led to the discovery of two proteins localised to the ER. These were found to be novel members of the prohibitin family and were named ER lipid raft protein (erlin)-1 and -2 [25]. This report is consistent with the observation that the Shiga toxin B-subunit remains associated with TX-100 DRMs during retrograde transport from the plasma membrane, and persists in its target compartment, the ER [42]. Also, PrPc, a GPI-anchored protein which is expressed in a wide spectrum of cell types including MECs [43], has been found to associate as an immature precursor to lipid raft already in the ER [44]. Another finding that has wide implications for the mechanisms of protein sorting and exit from the ER is the observation that apical, but not basolateral, secretory proteins are resistant to Tween 20 solubilisation during early stages in their biosynthesis in the ER [45]. The lipid composition of these DRMs is compatible with the presence of the corresponding lipid rafts in the ER. In the context of casein transport and casein micelle formation, we hypothesize that the membranous form of immature α_s1_-casein acts as a “nucleus” for casein association/aggregation in the ER for the targeting of the other caseins to the site of COP II vesicle formation and forward transport of the casein aggregates to the apical membrane. Amazingly, it has been demonstrated in both yeast and mammalian cells that loss of the GPI membrane anchor in marker proteins, and the resulting deficiency in association with the lipid microdomains in the ER, results in a reduced maturation rate and, therefore, slower transport of the proteins to the Golgi apparatus [46]–[48]. We also observed that, in the absence or with low amount of α_s1_-casein, there is reduction of the transport of the other caseins and their accumulation in distended ER cisternae [7]. The physiological relevance of this observation has not been clarified, but we suggest that the necessary interaction of α_s1_-casein with lipid microdomains may be at the center stage of the mechanism underlying the efficient transport and sorting of caseins.

The present study reveal that the insolubility of membrane-associated α_s1_-casein reflects its interaction with a cholesterol-rich detergent-resistant microdomain. We propose that the membrane-associated form of α_s1_-casein interacts with the lipid microdomain, or lipid raft, that form within the membranes of the ER, for packaging into COPII vesicles, efficient export from the ER, and forward transport and sorting in the secretory pathway of mammary epithelial cells.
